# Development of the Urogenital Microbiota in Healthy Beagle Puppies: A Longitudinal Comparison with the Dam

**DOI:** 10.3390/life16050841

**Published:** 2026-05-19

**Authors:** Marielle Somville, Bernard Taminiau, Virginie Gronsfeld, Sophie Egyptien, Flore Brutinel, Annick Hamaide, Georges Daube, Marie-Lys Van de Weerdt, Stefan Deleuze, Stéphanie Noël

**Affiliations:** 1 Department of Companion Animal Teaching and Clinical Practice, FARAH—Comparative Veterinary Medicine, Faculty of Veterinary Medicine, University of Liège, Avenue de Cureghem 1, 4000 Liège, Belgiums.deleuze@uliege.be (S.D.); stephanie.noel@uliege.be (S.N.); 2 Department of Food Sciences, Faculty of Veterinary Medicine, University of Liège, 10 Avenue de Cureghem, 4000 Liège, Belgium; bernard.taminiau@uliege.be (B.T.);; 3 Labforvet, 18 Rue Tienne aux Grives, 5300 Andenne, Belgium

**Keywords:** microbiota, urine, vagina, prostate, puberty, puppy, dogs

## Abstract

Characterizing the microbiota is essential to improve the understanding and management of urogenital disorders. Using 16S rDNA sequencing, this study investigated the urogenital microbiota, including urine, vaginal, and prostatic communities, in a litter of 10 healthy beagle puppies from 4 months of age until the completion of the first estrous cycle in females and 18 months in males. A further objective was to compare these microbial profiles with those of their dam. Significant differences were observed between urinary and genital microbiota in both sexes, evolving over time. Notably, in females, puberty and the first estrous cycle were associated with marked changes in the vaginal microbiota, outweighing individual variability. In contrast, urinary microbiota remained stable during female growth. In males, microbiota showed time-dependent and individual-specific progression, with distinct microbial communities identified in the urinary bladder and prostate. Shared genera between the dam and her offspring were observed, but inter-individual variability suggested a limited maternal influence. Further investigation is needed to clarify vertical transmission patterns. In conclusion, the urogenital microbiota of healthy dogs undergoes dynamic and distinct site-specific changes during early life.

## 1. Introduction

In recent years, interest in the microbiota has increased substantially. Before the advent of 16S rDNA gene sequencing, microbial communities were mainly investigated using culture-based techniques, which failed to detect a large proportion of resident microbiota [1]. More recently, the lack of standardized methodologies for microbiota research has prompted efforts to establish guidelines for critical steps such as sampling, storage, DNA extraction, and data analysis [2,3].

In humans, the resident microbiota of the bladder, prostate, and vagina have now been characterized under both physiological and pathological conditions. Alterations in the urogenital microbiota have been reported in women with urge urinary incontinence, interstitial cystitis, and bladder cancer, as well as in men affected by chronic prostatitis, benign prostatic hyperplasia, infertility, and bladder or prostate cancer [4,5]. It has been suggested that the establishment of the urinary microbiota during infancy may play a key role in the etiology and onset of urogenital diseases [6,7,8]. In newborns, microbial communities of the gut, skin, and oropharynx are influenced by maternal microbiota, mode of delivery, maternal diet, environmental exposure, and postnatal interactions such as colostrum intake and lactation [9,10,11]. Although in utero microbial colonization has been proposed in humans and animals, including reports of microbial DNA in the endometrium of cows and dogs and in foal amniotic fluid [12,13,14,15,16,17,18,19,20,21], this hypothesis remains controversial due to contradictory findings [22].

In dogs, characterization of the urogenital microbiota is still limited. A recent study reported that the meconium microbiota of vaginally delivered puppies resembled that of the maternal vaginal microbiota, whereas puppies born by cesarean section showed greater similarity to maternal oral and vaginal microbiota. Bacteria were isolated from 86.5% of meconium samples and 57% of placental samples, and puppies lacking detectable microbial communities exhibited slower growth rates [19]. These findings suggest that early microbial exposure may influence postnatal development in dogs.

The vaginal microbiota of adolescent girls differs from that of their mothers, with puberty-associated increases in *Lactobacillus* and other lactic-acid-producing bacteria [23]. Gerber et al. [7] suggested that as a lower urinary pH exerts a protective effect against lower urinary tract infection, hormonal changes at puberty may also be associated with maturation of the microbiota of the urinary tract. A previous study by Gronsfeld et al. [24] concluded that estrogenic impregnation influenced the genital microbiota in female dogs during the estrous cycle, similar to what has been described in women [25,26,27,28]. In a study comparing non-littermate prepubertal and postpubertal female dogs, vaginal microbiota was significantly different despite individual variations [18].

In healthy prepubertal boys, urine samples did not show a clear predominance of any bacterial genus across all individuals [8], but the microbiota differed from that previously described in adults in which *Staphylococcus* and *Corynebacterium* were predominant [29].

Conversely, children with neuropathic bladders displayed a predominance of the *Enterobacteriaceae* family in their urinary microbiota [30]. In men, the semen microbiota presents a modest similarity to the urinary microbiota. In cases of primary idiopathic infertility, patients harbored increased seminal ⍺-diversity, distinct β-diversity, and a higher abundance of seminal *Aerococus* [31].

For many years, urine was considered sterile [32]. However, advances in 16S rDNA gene sequencing have demonstrated the presence of distinct urinary microbial communities [33]. A recent study by Thassakorn et al. (2026) [34] shows that cat urine is dominated by *Proteobacteria*, with no effect of sex and minor age-related variations. In cases of feline idiopathic cystitis, a decrease in *Proteobacteria* and an increase in *Firmicutes* were described. Alterations in the urinary microbiota have also been described in cats with urethral obstruction or urolithiasis [35,36]. In humans, urinary microbiota composition varies according to physiological factors such as age and sex [37,38], and disease-associated dysbiosis has been reported in interstitial cystitis, characterized by a reduction in *Lactobacillus* [39,40].

To better understand the etiology and onset of urogenital disorders, it is essential to characterize both the genital and urinary microbiota in the early life and to follow its evolution during the prepubertal period in healthy dogs. To date, no data have been reported on the urinary, vaginal, and prostatic microbiota in a controlled cohort of puppies. Commensal bacteria may act in the homeostasis of the bladder function and as a barrier in the uroepithelium, outcompete uropathogens and produce antimicrobial compounds [31]. If epithelium integrity is compromised, microbial components may translocate into other host compartments. The present study aims to describe the urogenital microbiota of healthy male and female beagle dogs from puppyhood to adulthood, providing a foundation for future investigations into pathological conditions. The specific objectives of this study were as follows: (1) to describe the urogenital microbiota (bladder, vagina, and prostate) of healthy growing dogs up to 18 months of age in males and until the end of the first estrous cycle in females; (2) to compare the microbiota between urine and prostate in males, and between urine and vagina in females; and (3) to compare the urogenital microbiota between the dam and her puppies.

## 2. Materials and Methods

### 2.1. Study Population and Design

This prospective study was conducted on laboratory beagle dogs owned by the Veterinary Faculty of the University of Liège (ULiège ethical approval number: 20-2250; laboratory approval number: LA161012): a healthy beagle dam and her 10 healthy puppies from 4 to 18 months of age for the males and to the end of the first estrous cycle for the females. All puppies were born by vaginal delivery without complication. Dogs were housed together in the same kennel with wood shavings as bedding and received the same diet. The dam was fed a growth dry diet during lactation, which was also given to the puppies after weaning. From 12 months of age, all dogs received an adult maintenance dry diet. Environmental hygiene was maintained through daily removal of urine and feces and weekly replacement of bedding. Additionally, all dogs had daily access to an outdoor enclosure throughout the morning.

All dogs were weighed and had a complete physical examination, including a vaginal examination and a vaginal cytology for the female dogs and a preputial examination and a prostatic wash for the male dogs, prior to the study. Also, urine samples were obtained via cystocentesis, and urinalysis included a dipstick test, cytologic examination and bacteriological culture. Jugular blood samples were obtained from each dog for complete blood count and biochemistry. Dogs were included in this study if they showed no signs of systemic, vaginal, prostatic, or lower urinary tract disease and had not received antibiotics or anti-inflammatory drugs within the 30 days prior to sampling.

Samples were obtained from the female beagle dogs at 4, 6, 7, 8 and 9 months of age and at the following phases of the first estrous cycle: proestrus, estrus, diestrus, anestrus. Phases of the estrous cycle were identified via visual examination of the vulva, cytologic examination of a vaginal smear and measurement of plasma progesterone concentration (Automated Immunoassay analyzer 360, TOSOH). For the male beagle dogs, samples were obtained at 4, 6, 7, 8, 9, 10, 12 and 18 months of age. For the dam, the collection of samples started at 1 month postpartum then were collected in each phase of the next estrous cycle (proestrus, estrus, diestrus, and anestrus). Prior to each study time, each dog was weighed and a complete physical examination was performed.

In all dogs, urine was collected via prepubic cystocentesis after surgical skin asepsis with chlorhexidine soap and alcohol to minimize dermal microbiota contamination. Around 10 mL of urine was collected and divided into aliquots for routine analysis (specific gravity, dipstick with pH, and microscopic evaluation of the sediment), culture and 16S rDNA gene sequencing. For the female dogs, after placing a sterile (UV treatment for 30 min in a BSL2 Biohazard cabinet, optimale 12 cabinet, ADS laminaire, Le-pre-saint-gervais, France) otoscope cone beyond the vestibule, a swab moistened with sterile saline solution was passed through the speculum into the anterior vagina and was swabbed for 10 s to collect a genital sample. A second swab was collected for a bacteriological culture. Negative controls consisted of saline-moistened swabs passed through a sterile speculum. For male dogs, prostate samples were obtained by manual semen collection (third fraction of the ejaculate) or prostatic massage [41]. Samples were aliquoted for routine analysis, culture and 16S rDNA amplicon sequencing. Urine, vaginal, semen and prostatic wash samples were stored at −80 °C until DNA extraction.

### 2.2. Total Bacterial DNA Extraction and Amplicon Sequencing

DNA extraction, 16S rDNA library preparation, sequencing and informatics analysis were performed as previously described [42]. Briefly, total bacterial DNA was extracted from the vaginal swabs, the urine and the prostatic samples with a DNEasy Blood and Tissue kit (QIAGEN Benelux BV; Antwerp, Belgium) with an added bead beating step during lysis. Amplicon sequencing targeting V1V3 hypervariable regions of the 16S rDNA was performed using a MiSeq sequencer (Illumina; San Diego, CA, USA). Sequence reads were cleaned and processed using the MOTHUR software package v1.47 using SILVA v1.38.1 16S rDNA as the taxonomical reference database [43,44].

Cleaned sequences were binned into OTUs with 97% similarity threshold. Diversity and population analyses were performed on the genus level. To identify putative bacterial contaminants in vaginal swab samples, a multiple non-parametric Spearman Rho correlation test was performed between genus abundance and total bacteria load, determined by real-time PCR (see Supplementary Materials).

Molecular quantification of the bacterial content is based upon the quantitative amplification of the V2V3 hypervariable region of the 16S rDNA by real-time PCR [45]. The correlation was considered as significant for Rho values above 0.5 or below −0.5, with a *p*-value < 0.05. We also used the Decontam 1.28.0 package [46] in R to detect putative contaminants in the bacterial profiles, using the combined strategies and a threshold of 0.05 (see Supplementary Materials). The combined strategy used fluorescent dsDNA 16S V1V3 purified PCR product quantification and the profile of negative controls of the sequencing run. These negative controls were first mock samples undergoing the same DNA extraction process as true samples (respectively, sterile PBS and an unused swab for urine/prostate and vagina samples). The second kind of negative controls are the mock samples from the 16S V1V3 PCR.

### 2.3. Statistical Analysis

Good’s coverage index and ecological indicators, including the α-diversity (inverse Simpson’s index and Shannon index), bacterial richness (Chao1 index) and evenness (Simpson index-based measure), were calculated with MOTHUR v1.47. Alpha-diversity describes the within-sample microbial diversity, reflecting the number of taxa and their distribution in a given sample. Differences between groups were assessed using the non-parametric Friedman ANOVA test for repeated measures, followed by paired post hoc tests corrected with a two-stage linear step-up Benjamini–Hochberg procedure (q threshold = 0.05) with PRISM 9.0.

Beta-diversity was visualized with a Bray–Curtis dissimilarity matrix-based non-parametric dimensional scaling (NMDS) model using the vegan and vegan3d packages in R [47,48]. Beta-diversity describes the between-sample differences in microbial community composition, based on the presence/absence and/or relative abundance of taxa. Differences in beta-diversity between cycle phases were assessed with permutational multivariate tests Adonis2 and betadisper in R using the Bray–Curtis dissimilarity matrix. A *p*-value < 0.05 was considered significant. To assess time influence on the microbiota, the constrained ordination model (dbRDA) was computed for the three types of samples. The model and time fitting to the model were evaluated with ANOVA. Plots were created with ggplot2 package [49].

Differential population abundance between cycle phases was evaluated using a negative binomial Wald test in the DESeq2 R 1.48.2 package. Differences were considered significant if the corrected *p*-value was < 0.05 (Benjamini–Hochberg False Discovery Rate multi-testing correction). Time-associated abundance analysis was performed with LINDA command from the Microbiome Stat 1.4 package using time as a factor and Benjamini–Hochberg FDR to correct for multi-testing [50].

A matrix-correlation Mantel test [51] was performed with the Pearson, Spearman and Kendall tests to evaluate the correlation between vaginal and urinary microbiota.

## 3. Results

### 3.1. Study Population Characteristics

The study population included 10 beagle puppies (4 females and 6 males) born vaginally from the same dam. All dogs were clinically healthy throughout the study period and none had received antibiotics or anti-inflammatory drugs within 30 days prior to sampling. One male puppy was enrolled from 6 months of age because he had digestive surgeries at 4 and 5 months of age.

In male puppies, puberty was defined as the onset of spermatogenesis. Spermatozoa were first detected by cytological examination at a mean age of 8.6 months (range: 7–10 months). In female puppies, puberty was defined as the onset of the first estrous cycle. Vaginal cytology identified proestrus at a mean age of 10.8 months (range: 10–12.5 months).

### 3.2. Description of the Urogenital Microbiota (Bladder, Vagina and Prostate) of Healthy Growing Dogs—Male and Female

#### 3.2.1. Urinary Microbiota

**Figure 1:**
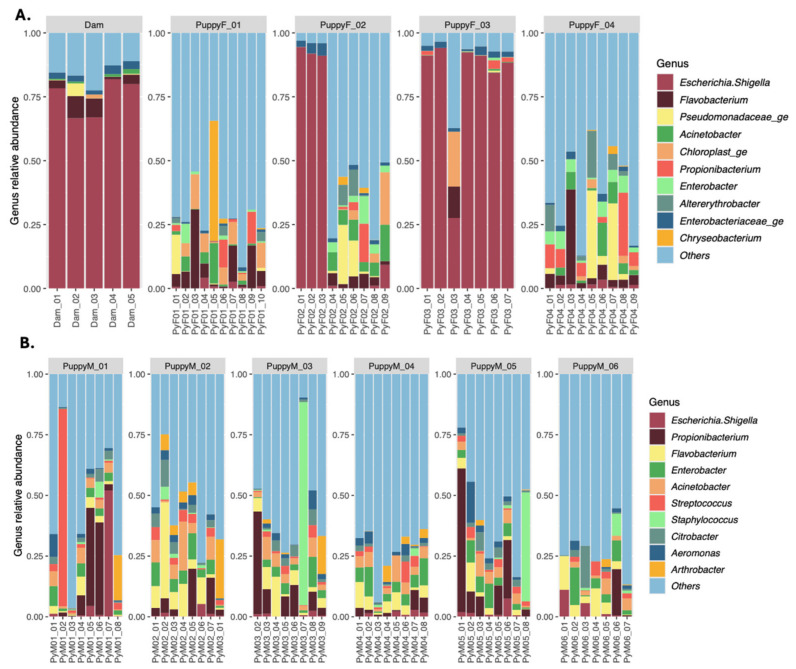
Genus relative count in (**A**) the urine samples from the dam (1-month postpartum, next proestrus, estrus, diestrus and anestrus) and from the female puppies (PuppyF) at 4, 6, 7, 8 and 9 months of age and at first proestrus, estrus, diestrus and anestrus, and in (**B**) male puppies (PuppyM) at 4, 6, 7, 8, 9, 10, 12 and 18 months of age.

The urinary microbiota of female puppies (Figure 1A) displayed a wide diversity but also pronounced inter-individual variability. Communities were characterized by a heterogeneous composition with no single genus consistently dominating across all individuals. Unclassified low-abundance taxa were grouped as “Others”.

Across females, major bacterial populations observed belonged to the genera *Flavobacterium*, *Acinetobacter*, *Propionibacterium*, and the *Escherichia*–*Shigella* group as well as populations from the *Pseudomonadaceae* family. Their relative abundances differed markedly between puppies and across time points. In one individual, *Escherichia*–*Shigella* predominated in the first samples before transitioning to a more diverse community. Another puppy showed a constant *Escherichia*–*Shigella* group predominance, resembling the maternal urinary profile. The other females exhibited more diverse and unstable communities with fluctuating dominant genera. Taken together, the female urinary microbiota showed high diversity but no clear convergence pattern over time.

In male puppies, the urinary microbiota (Figure 1B) also showed pronounced inter-individual and temporal variability. Communities were composed of fluctuating combinations of genera such as *Flavobacterium*, *Propionibacterium*, *Enterobacter*, *Acinetobacter*, and the *Escherichia*–*Shigella* group. No single taxon was consistently dominant across all males. After exclusion of unclassified taxa, the male urinary microbiota remained characterized by instability over time and recurrent detection of a limited set of genera with variable relative abundances.

Comparison between sexes indicated that both male and female puppies harbored diverse and unstable urinary microbiota during growth (Figure 1). However, microbial community composition differed significantly between sexes, with distinct bacterial profiles and dominant taxa identified in males and females (*p* < 0.001).

#### 3.2.2. Vaginal Microbiota

**Figure 2:**
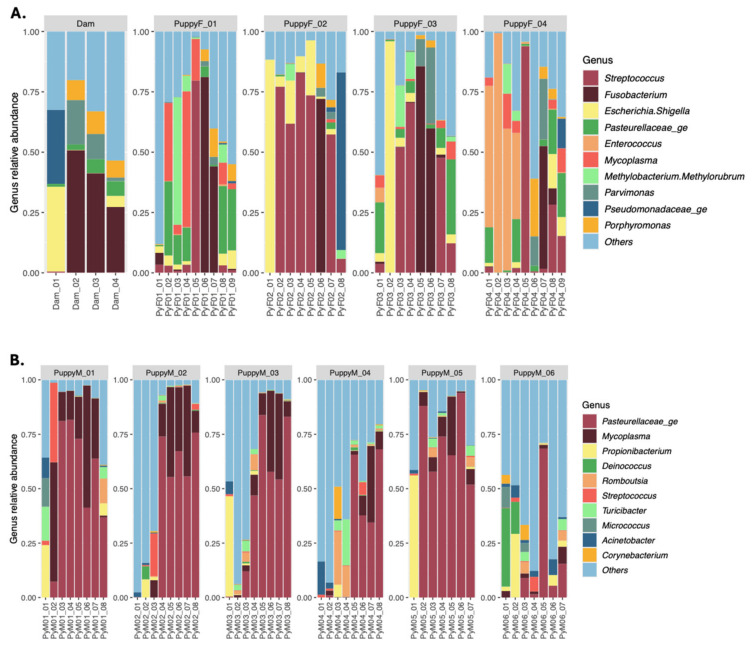
Genus relative count in (**A**) the vaginal samples from the dam (1-month postpartum, next proestrus, estrus, diestrus and anestrus) and from the female puppies (PuppyF) at 4, 6, 7, 8 and 9 months of age and at first proestrus, estrus, diestrus and anestrus and in (**B**) the prostate samples of the male puppies (PuppyM) at 4, 6, 7, 8, 9, 10, 12 and 18 months of age.

The vaginal microbiota of female puppies (Figure 2A) was heterogeneous and dynamic, with marked inter-individual differences. Communities were mainly composed of *Streptococcus*, *Fusobacterium*, and *Enterococcus genera*, as well as the *Escherichia*–*Shigella* group. Some puppies showed alternating dominance patterns across sampling times, whereas others were characterized by repeated predominance of a single genus, such as *Streptococcus* or *Enterococcus*.

#### 3.2.3. Prostatic Microbiota

The prostatic microbiota of male puppies (Figure 2B) was dominated by a limited number of genera. Five of the six puppies displayed relatively homogeneous profiles dominated by members of the *Pasteurellaceae* family, with intermittent increases in *Mycoplasma*. Minor taxa such as *Propionibacterium* and *Streptococcus* were sporadically detected. In contrast, one puppy showed a more heterogeneous and unstable prostatic profile.

### 3.3. Comparison of the Microbiota Between Urine and Vagina in Female Puppies

**Figure 3:**
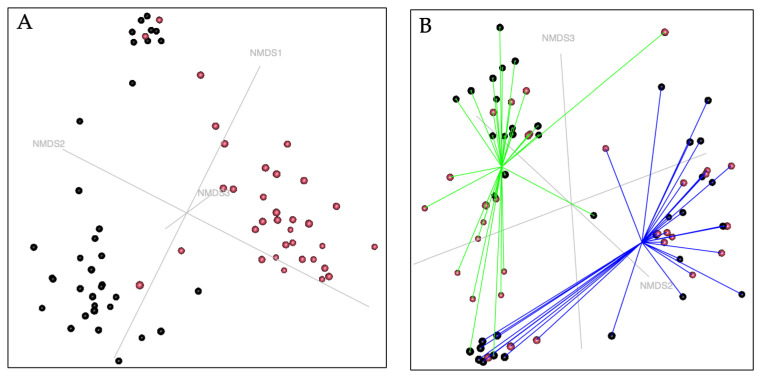
NMDS ordination of urinary and vaginal microbiota in female puppies (k = 4, stress = 0.12). Non-metric multidimensional scaling (NMDS) model based upon a Bray–Curtis dissimilarity matrix of the female puppies microbiota. (**A**) Urine samples are colored in black and vagina samples are colored in red. (**B**) Samples are colored by pubertal status (black = prepubertal, red = pubertal). Samples are joined by vectors colored by the type of sample (blue = urine sample, green = vaginal sample) to the centroid of the group.

Non-metric multidimensional scaling (NMDS) analysis revealed a clear separation between urinary and vaginal microbiota in female puppies (Figure 3), indicating distinct community structures between the two anatomical sites (*p* = 0.001). This compartment-specific clustering was observed in both prepubertal and pubertal samples, with increased dispersion of vaginal samples after puberty, suggesting higher inter-individual variability.

**Figure 4:**
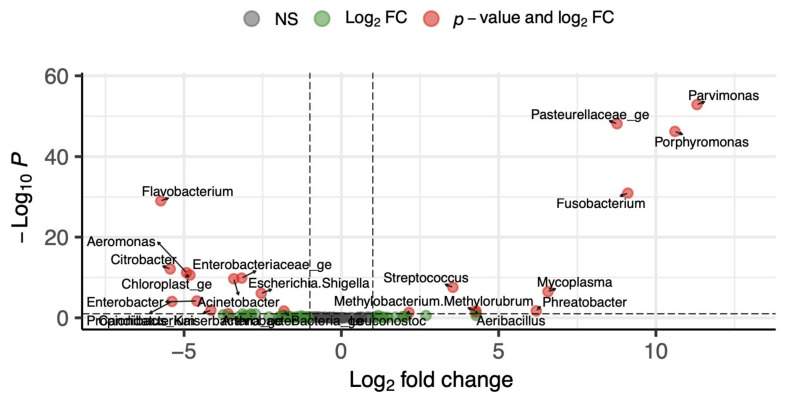
Volcano plot of differential bacterial abundance analysis performed with DeSEQ2, between urine and vagina female puppies. The x-axis shows log_2_ fold change (urine vs. vagina) and the y-axis represents the negative value of the log10 transformed adjusted *p*-value. DeSEQ2 test was performed using a BH FDR with q = 0.05. log_10_ *p*. Points are colored by significance (gray: non-significant; green: log_2_ FC only; red: *p*-value and log_2_ FC).

Differential abundance analysis further supports these findings (Figure 4). Vaginal samples were enriched in anaerobic genera, including *Porphyromonas*, *Parvimonas*, *Fusobacterium*, and members of the *Pasteurellaceae* family. In contrast, urinary samples were enriched in *Flavobacterium*, *Aeromonas*, *Citrobacter*, and *Enterobacter*. These results demonstrate clear site-specific microbial signatures in female puppies.

### 3.4. Comparison of the Microbiota Between Urine and Prostate in Male Puppies

**Figure 5:**
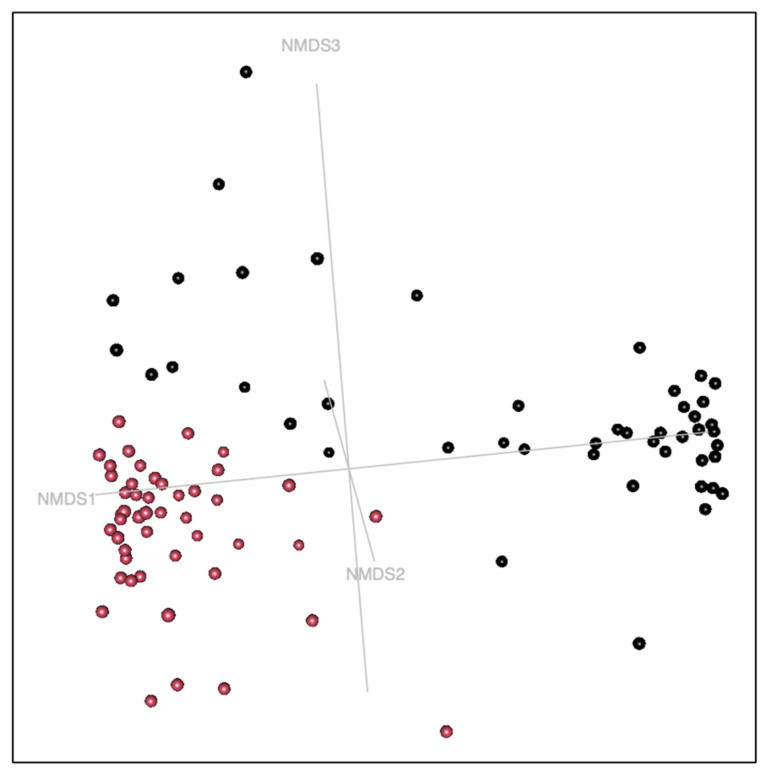
Non-metric multidimensional scaling (NMDS) model of the male puppies’ microbiota samples, based upon a Bray–Curtis dissimilarity matrix (NMDS model k = 4, stress = 0.09). Samples are colored by the nature of the sample (black = prostate, red = urine).

**Figure 6:**
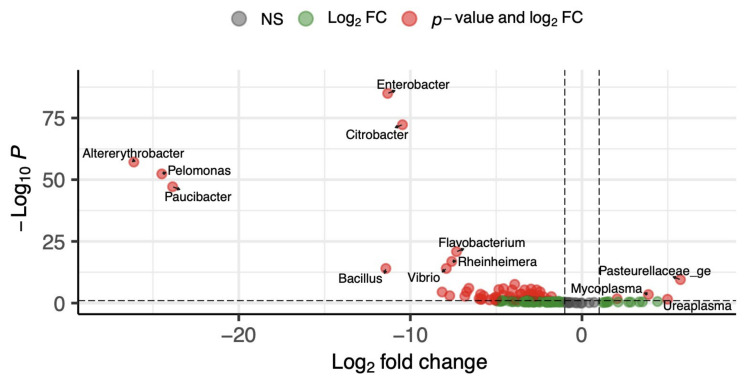
Volcano plot of differential bacterial abundance analysis performed with DeSEQ2 between urine and prostate in male puppies. The x-axis shows log_2_ fold change (urine vs. prostate) and the y-axis represents the negative value of the log10 transformed adjusted *p*-value. DeSEQ2 test was performed using a BH FDR with q = 0.05. log_10_ *p*. Points are colored by significance (gray: non-significant; green: log_2_ FC only; red: *p*-value and log_2_ FC).

In male puppies, NMDS analysis also showed a clear separation between urinary and prostatic microbiota (Figure 5), indicating distinct bacterial community structures between the two compartments (*p* = 0.001). In addition, differential abundance analysis (Figure 6) identified several taxa with different relative abundances. The genera *Enterobacter* and *Citrobacter* were enriched in the prostate, whereas *Ureaplasma* and *Mycoplasma*, as well as the *Pasteurellaceae* family, were more abundant in urine. These findings further support the presence of compartment-specific microbiota in male dogs.

### 3.5. Temporal Evolution of the Microbiota During Growth

#### 3.5.1. Urinary Microbiota

**Figure 7:**
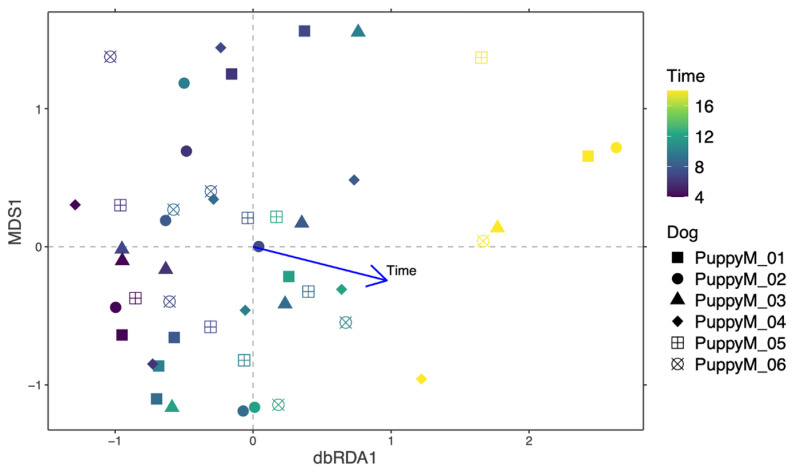
Temporal structuring of the urine microbiota in male dogs. Bray–Curtis distance-based redundancy analysis (dbRDA) of the urine microbiota in male puppies during their growth. Each point represents one sample, colored according to sampling time and shaped by individual dog. The arrow indicates the direction and strength of the time factor, fitted to the domain.

**Figure 8:**
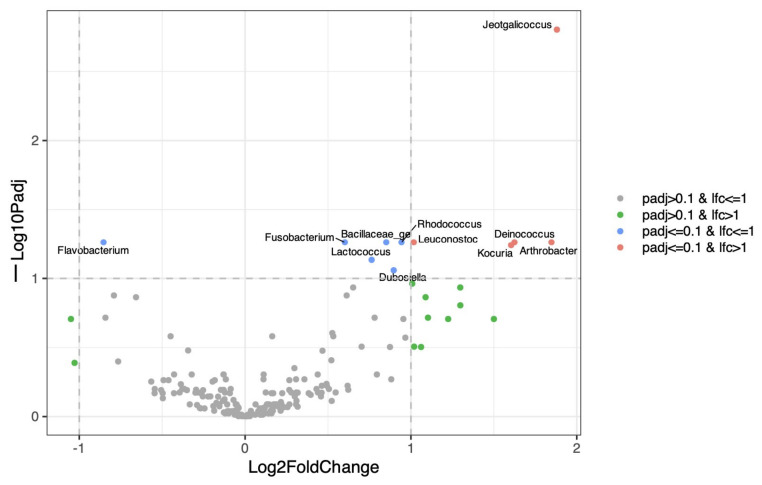
Limited temporal changes in the male urinary microbiota. Volcano plot of time-associated differential abundance analysis of the male urinary microbiota using linear mixed-effects models (LINDA) with dog as a random effect. The x-axis shows log2 fold change associated with time and the y-axis shows −log10 adjusted *p*-values. Only a small number of taxa display modest temporal changes, mainly involving environmental or skin-associated genera. This indicates a limited maturation of the male urinary microbiota during growth.

In males, distance-based redundancy analysis (dbRDA) revealed a modest but significant effect of time on the urinary structure (*p* = 0.001) (Figure 7). An overlap between time points and high inter-individual variability were observed, indicating heterogenous temporal dynamics. Only a limited number of taxa showed significant temporal changes (Figure 8).

**Figure 9:**
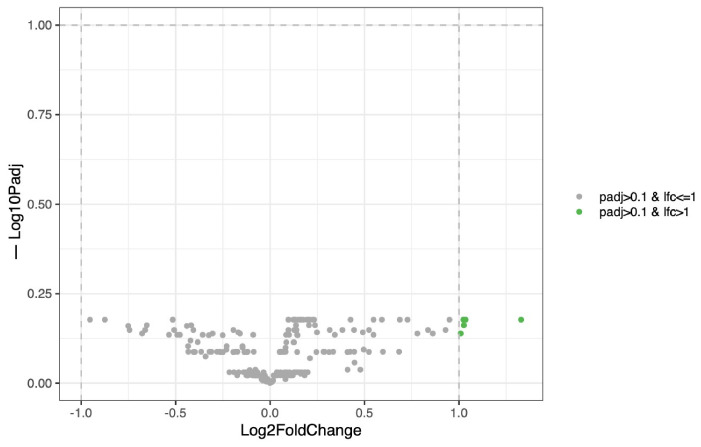
Time-related effects on the urinary microbiota in female puppies. Volcano plot showing the effect of time on urinary bacterial taxa in female puppies, assessed using a mixed-effects model (LINDA). The x-axis indicates the log2 fold change in relative abundance over time, while the y-axis shows the −log10 adjusted *p*-value. No taxa exhibited statistically significant temporal variation after multiple testing correction (padj ≤ 0.1), suggesting a stable urinary microbiota throughout growth.

For the female group, we did not find significant temporal variation across growth (*p* = 0.15). No taxa demonstrated significant changes in abundance over time, supporting a high degree of stability in the urinary microbiota (Figure 9).

#### 3.5.2. Vaginal Microbiota

**Figure 10:**
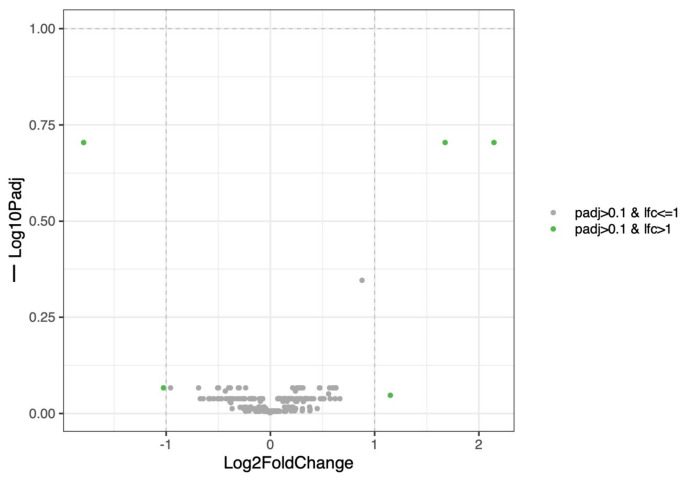
Time-related effects on the vaginal microbiota in female puppies. Volcano plot of time-associated differential abundance analysis of the female vaginal microbiota using linear mixed-effects models (LINDAs). The x-axis shows log2 fold change associated with time and the y-axis shows −log10 adjusted *p*-values. No taxa reached statistical significance after correction for multiple testing (padj ≤ 0.1), indicating no significant time-dependent changes in vaginal microbiota composition during the study period.

Mixed-effects modeling revealed no statistically significant time-dependent shifts in vaginal microbiota composition across the study period (*p* > 0.05) (Figure 10). A limited number of taxa were identified, and changes did not reach statistical significance. However, comparison between the first sampling point and the final sampling at the end of the first estrous cycle revealed significant differences in microbial composition (*p* = 0.001). Also, significant differences were observed depending on the cycle stage (*p* = 0.001), particularly between proestrus/estrus and diestrus/anestrus.

#### 3.5.3. Prostatic Microbiota

**Figure 11:**
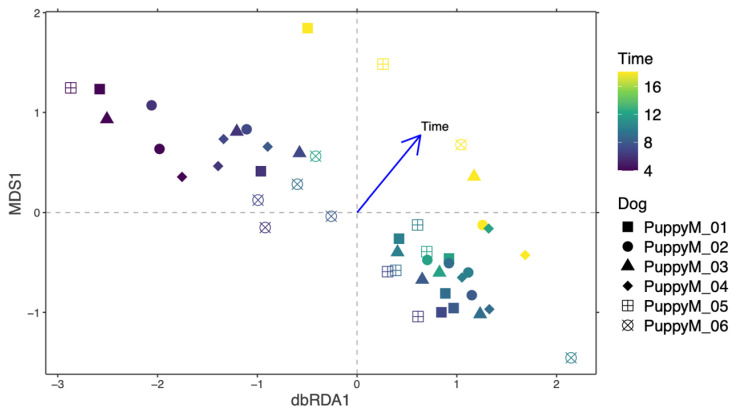
Temporal structuring of the prostatic microbiota in male dogs. Bray–Curtis distance-based redundancy analysis (dbRDA) of the prostatic microbiota in male puppies during their growth. Each point represents one sample, colored according to sampling time and shaped by individual dog. The arrow indicates the direction and strength of the time factor, fitted to the domain.

**Figure 12:**
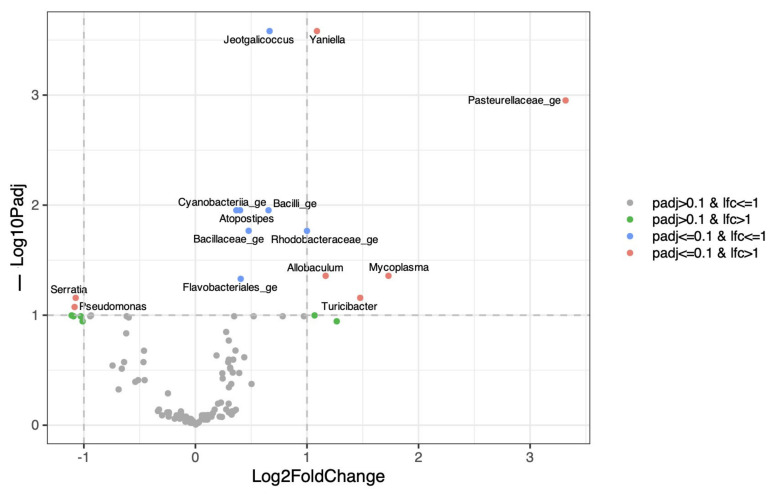
Time-associated taxa in the male prostatic microbiota. Volcano plot showing differential abundance analysis of prostatic bacterial taxa using linear mixed-effects models with time as a fixed effect and dog as a random effect (LINDAs). The x-axis represents log2 fold change associated with time, and the y-axis represents −log10 adjusted *p*-values. Taxa significantly increasing or decreasing over time are highlighted, indicating bacterial groups contributing to the temporal maturation of the prostatic microbiota.

In contrast, dbRDA analysis demonstrated a significant effect of time on the structure of the prostatic microbiota in male puppies (*p* = 0.002) (Figure 11), which outweighed inter-individual variability. Differential abundance analysis showed that the *Pasteurellaceae family*, as well as *Mycoplasma* and *Ureaplasma*, increased over time, whereas environmental or opportunistic taxa such as *Pseudomonas*, *Acinetobacter*, and *Enterobacter* were mainly detected at earlier time points (Figure 12).

### 3.6. Comparison of the Urogenital Microbiota Between the Dam and Her Puppies

**Figure 13:**
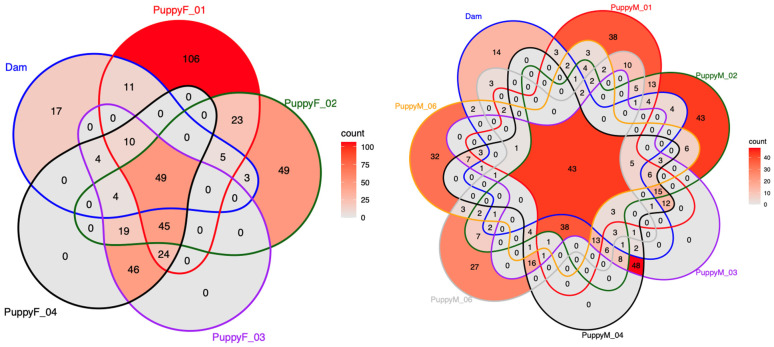
Venn diagram showing the number of shared and unique bacterial genera detected in urine samples from the dam and her female ((**left**), PuppyF) and male ((**right**), PuppyM) puppies. Each ellipse represents one individual, and numbers indicate the count of genera unique to, or shared between, individuals. Color intensity reflects the total number of genera detected per sample.

**Figure 14:**
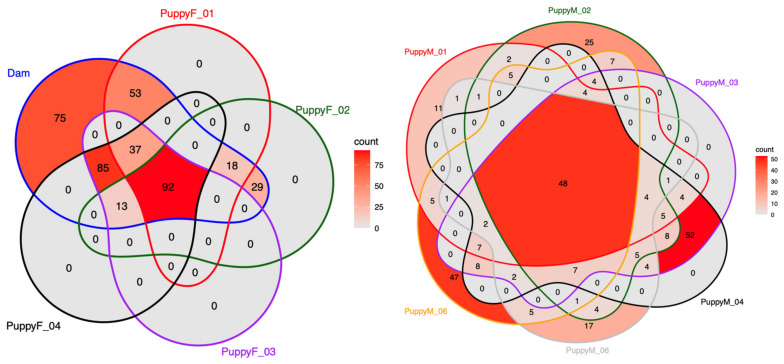
Venn diagrams showing the number of shared and unique bacterial genera detected in vaginal samples from the dam and her female puppies ((**left**), PuppyF) and in prostatic samples from male puppies ((**right**), PuppyM). Each ellipse represents one individual, and numbers indicate the count of genera unique to, or shared between, individuals. Color intensity reflects the total number of genera detected per sample.

Venn diagram analyses revealed the presence of shared bacterial genera between the dam and her puppies in both urinary and genital compartments (Figure 13 and Figure 14). In urine, 50 genera were shared between the dam and female puppies and 44 between the dam and male puppies. However, each puppy also harbored a substantial number of unique taxa. The dam’s urinary microbiota was highly uniform and dominated almost exclusively by the group *Escherichia*–*Shigella* across all sampling points, whereas puppies exhibited more diverse, low-abundance, and fluctuating urinary profiles (Figure 1).

In the vaginal compartment, female puppies shared several genera with the dam, particularly during early life. Comparing the first samples of female puppies with the mother, common populations were *Streptococcus*, *Enterobacter*, and *Acinetobacter*. Later, increasing diversification was observed with age.

In male puppies, there was only a limited overlap in bacterial genera. A small number of taxa, including *Enterobacter*, *Acinetobacter,* and *Streptococcus*, were occasionally detected in both compartments, mainly at early time points. Dominant genera differed markedly between matrices, with the dam’s urine being largely dominated by the *Escherichia–Shigella* group, whereas the prostatic microbiota of puppies was primarily characterized by *Mycoplasma* and the *Pasteurellaceae* family (Figure 2).

## 4. Discussion

To the authors’ knowledge, this is the first study to longitudinally characterize the urinary, vaginal, and prostatic microbiota in healthy growing dogs, and to compare these profiles with those of their dam. We followed a controlled cohort of beagle puppies from early life to puberty and adulthood, and our results indicate that the canine urogenital microbiota is not only site-specific but also dynamically shaped by sex and developmental stage. Urinary microbiota exhibited limited maturation, with stability particularly evident in females, while males showed a modest but significant temporal effect. In contrast, genital microbiota underwent more pronounced changes during growth and around puberty. The difference in follow-up duration reflects biological differences between sexes. In females, the vaginal microbiota is influenced by hormonal fluctuations across the estrous cycle, justifying follow-up until completion of the first cycle. In males, where microbiota changes more gradually, follow-up was set at 18 months of age, corresponding to young adulthood.

The urinary microbiota of our puppies was diverse and highly variable between individuals, confirming that urine is not sterile even early in life. In both sexes, no dominant bacterial genus was detected across all individuals, and temporal fluctuations were common. *Flavobacterium*, *Acinetobacter*, *Propionibacterium*, *Escherichia*–*Shigella* group, and *Pseudomonadaceae* were recurrently detected, although their relative abundances differed markedly. Our results were also partially consistent with studies from Burton et al. [33] and Hu et al. [41], describing *Flavobacterium*, *Enterobacter*, and *Rheinheimera* as the dominant genera, as well as *Escherichia*–*Shigella* group.

The inter-individual variability is consistent with previous studies in other species, in which the urobiome was more individualized than age-related. For example, the recent study of Thassakorn et al. (2026) showed that cat’s urine was dominated by *Proteobacteria*, but no effect of sex was found, and minor age-related variations were observed [34]. On the other hand, human studies showed that urinary microbiota composition varies according to physiological factors such as age and sex [37,38].

Despite individual variability, the female urinary microbiota appeared remarkably stable over time, with no clear distinction between the two stages of life. No significant temporal effect was detected across growth in our study, suggesting that it may rapidly reach a steady-state configuration under physiological conditions. Similar stability has been reported in adult cycling bitches [24] and women [38], with hormonal fluctuations exerting a minor effect on urinary microbial communities.

In contrast, a modest but significant time effect was observed in male puppies, although inter-individual variability remained the predominant source of variation. Similarly, urine samples of healthy prepubescent boys did not show a clear predominance of any particular bacterial genus [8], but the microbiota differed from that described in adults, where *Staphylococcus* and *Corynebacterium* predominated [29].

The male urobiome was mainly composed of *Flavobacterium*, *Propionibacterium*, *Enterobacter*, and *Acinetobacter* in varying proportions, as well as the *Escherichia*–*Shigella* group. Regarding temporal variations, only a small number of taxa, such as *Jeotgalicoccus*, *Arthrobacter*, and *Kocuria*, showed moderate differences. Isolated samples from the third and fifth male puppies exhibited an unusual dominance of *Staphylococcus*, a genus commonly associated with skin or environment microbiota. This result could reflect a sample handling or collection contamination rather than a true shift in the urinary composition given the absence of similar patterns in adjacent time points and in the other puppies.

The limited maturation observed may reflect the continuous exposure of the bladder to urine flow, together with intermittent microbial contamination from the environment and adjacent anatomical sites, including the perineal skin and enteric microbiota. Similar patterns, in which inter-individual variability outweighs age-related effects, have been reported in human pediatric cohorts [8] and in adult canine urobiome studies [33].

The method of sampling is a critical factor in microbiota studies. Given the low microbial biomass of urine, these samples are highly susceptible to contamination at every step of the analytical work, as is the vaginal tract [2,3]. Therefore, careful interpretation of detected taxa is essential, particularly for genera commonly associated with the environment or skin.

The vaginal microbiota of female puppies showed greater temporal variability than the urinary microbiota and was characterized by heterogeneous community structures across individuals. Genera recurrently detected in the canine vagina included *Streptococcus*, *Enterococcus*, *Fusobacterium, Mycoplasma*, the group of *Escherichia*–*Shigella*, and the *Pasteurellaceae* family. *Methylobacterium*, which appeared in a single peak in only one sample, is a well-recognized environmental contaminant in low-biomass microbiota studies and is not considered a typical component of the mammalian vaginal microbiota [52].

This diversity is consistent with previous descriptions of the canine vaginal microbiota, which, unlike the human vagina, is not dominated by a single genus such as *Lactobacillus* [53]. A study from Spanoghe et al. [2] found *Porphyromonas*, *Parvimonas* and *Fusobacterium* to be the main bacteria present in the canine vagina of six healthy bitches in estrus. They also noted that microbial compositions vary between studies and hypothesized that these discrepancies are driven by differences in sampling procedures, sample storage conditions, and methodological approaches.

Although no statistically significant global time effect was identified, marked compositional changes were observed around puberty, particularly between proestrus/estrus and diestrus/anestrus. These variations are consistent with previously reported microbiota shifts associated with estrous cycle phases [24]. They were characterized by a reduction in community heterogeneity and the emergence of profiles resembling those described in adult bitches. Together, these findings suggest that puberty represents a key modulatory period, with estrogenic influence likely playing a major role in shaping the vaginal microbiota. Estrogen-driven modifications of the vaginal epithelium, mucus production, and local immune responses are well documented and could contribute to alterations in microbial composition. Similar shifts have been described in women and adolescent girls with an increase in *Lactobacillus* at puberty, as well as in adult cycling bitches [23,24,25,26,27,28,41]. In a study comparing non-littermate prepubescent and cycling bitches, vaginal microbiota was significantly different despite individual variations [18]. In this context, pubertal status appeared to exert a stronger influence on vaginal microbial composition than individual identity, thereby reinforcing the role of estrogen fluctuations in modulating the vaginal microbial ecosystem.

To date, data on the physiological prostatic microbiota in dogs remain scarce. In our male puppies, the prostatic microbiota was dominated by a limited number of taxa, primarily members of the *Pasteurellaceae* family and *Mycoplasma*. In contrast, one puppy exhibited a more heterogeneous and unstable prostatic profile. Members of the *Weeksellaceae* family are not classically described as part of the urogenital microbiota in mammals and are often associated with environmental sources, suggesting transient exposure or possible contamination.

Although notable inter-individual variability was found, the prostatic microbiota showed a more significant time effect and a consistent temporal shift during growth. Early samples were characterized by the presence of more diverse and potentially environmental or opportunistic genera, whereas later time points showed an increase in taxa previously listed. This progressive shift suggests a maturation process toward a more specialized microbial community. Similar compartment-specific microbiota and age-related changes have been described in humans [38,54].

Clear differences were observed between urinary and genital microbiota in both sexes, even at early developmental stages, as evidenced by β-diversity analyses. Microbial overlap between compartments was limited, and each site exhibited distinct temporal dynamics and compositional characteristics. In males, the prostatic microbiota was composed of a relatively small set of specific taxa, including members of the *Pasteurellaceae* family and *Mycoplasma*, whereas the bladder harbored a more diverse, dynamic microbial community. In both sexes, the bladder is regularly flushed during urination and exposed to a dynamic environment, whereas the vagina and prostate represent more stable and specialized sites. Distinct epithelial structures, pH, glandular secretions, and local immune defenses likely contribute to maintaining microbial communities in each compartment. In addition, in males, the prostate is an encapsulated gland with a relatively impermeable hemato-prostatic barrier, which may further limit microbial influx from adjacent sites [18,24,33].

These findings highlight a strong anatomical site specificity, consistent with previous canine and human studies. Taken together, these results suggest that both anatomical structure and physiological processes play a role in shaping compartment-specific microbiota, and that similar patterns of site-specific organization are observed across species [31,38,54].

Comparison of the urogenital microbiota between the dam and her puppies revealed the presence of shared bacterial genera in both urinary and genital compartments. However, each puppy also harbored a distinct set of taxa, and overall overlap was limited, indicating that early environmental exposure rapidly influences the urogenital microbiota. Also, the individual effect remained stronger than environmental factors, as differences were observed between puppies raised under identical conditions and diet.

The dam’s urinary microbiota was highly uniform and dominated by the *Escherichia–Shigella* group, whereas puppies exhibited more diverse and fluctuating communities. Two female puppies displayed more adult-like profiles: in one, the predominance of *Escherichia*–*Shigella* was transient before transitioning toward a more diverse community, while in the other it persisted throughout the study period. Such patterns were not observed in the remaining females, whose urobiomes were already diverse by 4 months of age. This could suggest that the early predominance of the *Escherichia*–*Shigella* group may be temporary and may eventually be outweighed by individual-specific and environmental factors. In contrast, *Escherichia*–*Shigella* was present at low abundance in male puppies.

In the genital compartment, particularly in females, puppies shared several genera with the dam, including *Streptococcus*, *Enterobacter*, and *Acinetobacter*, indicating a potential maternal contribution to initial microbial exposure. In males, prostatic microbiota showed limited shared taxa and greater inter-individual variability.

Early microbial colonization occurs rapidly after birth, as reported by Perez-Muñoz et al. [55], and may even begin during fetal life [12,13,14,15,16,17,18]. Del Carro et al. [56] showed that each dam–litter unit harbored a distinct fecal microbiota at day 0, day 2, day 30 and day 60 postpartum, with greater similarity within units than between them, supporting an important maternal role in early gut colonization, in agreement with Vilson et al. [57]. However, the study did not assess microbiota evolution beyond early life. Hand et al. [58] further reported that closely related dogs shared more similar microbiota profiles than unrelated dogs of the same breed. Guard et al. [59] observed a progressive increase in fecal microbial diversity from 2 to 56 days of age, with compositions significantly differing from those of the dams. More recently, the meconium microbiota of vaginally delivered puppies was found to resemble the maternal vaginal microbiota, whereas cesarean-delivered puppies showed greater similarity to maternal oral and vaginal microbiota. Moreover, puppies without detectable microbial communities exhibited slower growth rates [19].

Such exposure could occur during parturition, nursing, or close postnatal contact. Nevertheless, increasing diversification with age and marked inter-individual variability suggest that environmental and individual-specific factors rapidly outweigh any early potential maternal influence. These observations are compatible with a possible maternal contribution but do not provide direct evidence of vertical transmission. In addition, our study was not specifically designed to investigate this question. The use of a single litter and the absence of immediate postnatal sampling limit the ability to draw firm conclusions regarding such mechanisms. The environment is known to have an early influence on puppies’ microbiota.

This study has several limitations. First, as previously underlined, the sample size was small and included a single litter, which limits the generalizability of the findings. However, this cohort of ten puppies (six males and four females) from the same dam provided a unique opportunity to investigate microbiota development under controlled conditions, thereby reducing environmental and maternal variability compared to studies involving multiple litters. This homogeneous setting, including shared genetic background, housing conditions, and diet, allowed for a consistent baseline and limited potential confounding factors. Nevertheless, such standardization may not reflect the diversity of environmental and nutritional conditions encountered in the general canine population. Previous studies in humans have shown that housing conditions and diet can significantly influence the urogenital microbiota [26,27,28]. In dogs, weaning represents a critical period for microbial shifts, as the transition from maternal milk to solid food induces predictable changes in microbial communities, including those of the urogenital tract [11,60]. Future studies including multiple litters raised under varied environmental and nutritional conditions would therefore be valuable to extend these findings. Nevertheless, the high inter-individual variability observed in this study suggests that establishing a robust baseline is an important first step.

Second, urine, vaginal, and prostatic samples are low-biomass matrices susceptible to contamination. Despite controlled conditions, our vaginal sampling method using an otoscope cone may have increased the risk of contamination from the caudal vagina, as discussed by Spanoghe et al. [2], who recommended the use of a double-guarded sterile swab reaching the cranial vagina, as well as wearing sterile gloves to minimize contamination.

In addition, birth is not a sterile process. Bacterial colonization begins immediately after exposure to the external environment [10,19], making it difficult to completely rule out environmental contamination. The study design did not assess very-early-life colonization, as sampling began at four months of age rather than immediately after parturition. The dam was sampled at one month postpartum, and one male puppy was only included from six months of age due to previous digestive surgeries. Therefore, no direct early neonatal comparison or evaluation of immediate maternal transmission was possible. Studies from birth to adulthood with the objective of identifying the healthy microbiota composition are difficult to realize due to environmental factors, individual genetic makeup, and exposure to pathogens that are confounding factors, as described in humans [10,13,55].

## 5. Conclusions

This longitudinal study provides reference data on the development of the urogenital microbiota in healthy growing dogs from 4 months old to adulthood. By analyzing urinary, vaginal, and prostatic compartments, we demonstrate that the canine urogenital microbiota is strongly site-specific and influenced by sex and developmental stage. While the urinary microbiota showed marked inter-individual variability and limited maturation signals, genital microbiota underwent more pronounced age-related changes, especially around puberty. Shared genera between the dam and her offspring were observed, but inter-individual variability suggests a limited maternal influence.

These findings provide a physiological baseline for future studies investigating urogenital disorders in dogs, such as urinary tract infections, incontinence, vaginitis or prostatitis, and potentially neoplastic diseases. Given that pathological microbial shifts are implicated in various disorders in humans, understanding normal microbiota development is essential to identify dysbiosis-associated patterns and improve diagnostic and therapeutic strategies in veterinary medicine. Also, these findings should be interpreted within the context of a controlled cohort of beagle dogs and may not be directly generalizable to other breeds or environmental conditions.

Future research involving larger and more diverse populations, earlier postnatal sampling, and longitudinal follow-up will be needed to clarify host–microbiota interactions, maternal influence and environmental factors.

## Data Availability

The sequencing data generated in this study are publicly available in the NCBI Sequence Read Archive (SRA) under BioProject accession numbers PRJNA1082225 (dam samples) and PRJNA1443666 (puppies’ samples).

## Supplementary Materials

The following supporting information can be downloaded at: https://www.mdpi.com/article/10.3390/life16050841/s1, Excel file: Decontam.output; R file: Analysis output.

## Abbreviations

The following abbreviations are used in this manuscript:

16S rDNA	16S ribosomal DNA
dbRDA	distance-based redundancy analysis
DNA	deoxyribonucleic acid
NMDS	non-metric multidimensional scaling
OTU	operational taxonomic unit
PCR	polymerase chain reaction
RNA	ribonucleic acid
UV	ultraviolet
